# An aptamer agonist of the insulin receptor acts as a positive or negative allosteric modulator, depending on its concentration

**DOI:** 10.1038/s12276-022-00760-w

**Published:** 2022-04-27

**Authors:** Na-Oh Yunn, Jimin Lee, Hye Sun Lee, Eun Ju Oh, Mangeun Park, Seongeun Park, Seo Yeon Jin, Euisu Shin, Jo woon yi Lee, Youndong Kim, Sun Sik Bae, Sung Ho Ryu

**Affiliations:** 1 Postech Biotech Center, Pohang University of Science and Technology (POSTECH), Pohang, 37673 Republic of Korea; 2 The Department of Life Sciences, Pohang University of Science and Technology (POSTECH), Pohang, 37673 Republic of Korea; 3 Department of Pharmacology, School of Medicine, Pusan National University, Yangsan, 50612 Republic of Korea; 4 Aptamer Sciences, Inc., Seongnam, 13605 Republic of Korea

**Keywords:** Cell signalling, DNA and RNA

## Abstract

Aptamers are widely used as binders that interact with targets with high affinity or as inhibitors of the function of target molecules. However, they have also been used to modulate target protein function, which they achieve by activating the target or stabilizing its conformation. Here, we report a unique aptamer modulator of the insulin receptor (IR), IR-A62. Alone, IR-A62 acts as a biased agonist that preferentially induces Y1150 monophosphorylation of IR. However, when administered alongside insulin, IR-A62 shows variable binding cooperativity depending on the ligand concentration. At low concentrations, IR-A62 acts as a positive allosteric modulator (PAM) agonist that enhances insulin binding, but at high concentrations, it acts as a negative allosteric modulator (NAM) agonist that competes with insulin for IR. Moreover, the concentration of insulin affects the binding of IR-A62 to IR. Finally, the subcutaneous administration of IR-A62 to diabetic mice reduces blood glucose levels with a longer-lasting effect than insulin administration. These findings imply that aptamers can elicit various responses from receptors beyond those of a simple agonist or inhibitor. We expect further studies of IR-A62 to help reveal the mechanism of IR activation and greatly expand the range of therapeutic applications of aptamers.

## Protein function: subtle surprises from controlling molecules

Studying how an aptamer, a short section of RNA or DNA, affects the interaction of insulin with its membrane receptor protein offers further insights into aptamers in general. Aptamers can bind with high specificity and affinity to many target molecules, and affect the activity of many proteins. Researchers in South Korea led by Sun Sik Bae at Pusan National University and Sung Ho Ryu at Pohang University of Science and Technology explored the interaction of the aptamer IR-A62 with the membrane protein that binds to and responds to insulin. Whether IR-A62 activated or inhibited insulin’s interaction and effects depended on both the aptamer and insulin concentrations. While increasing understanding of the insulin receptor protein, investigating this subtly variable effect could more generally refine and expand the use of aptamers in medicine.

## Introduction

Aptamers are reagents that bind to a variety of targets, ranging from small molecules to cultured cells, with high affinity and specificity^1^. They are single-stranded oligonucleotides that are isolated by the in vitro selection process Systematic Evolution of Ligands by Exponential Enrichment (SELEX) from random oligonucleotide libraries^2,3^. Short oligonucleotides can fold into unique tertiary conformations, allowing aptamers to interact with their targets by specifically wrapping or fitting into the surface structures of the target molecules^4^.

The development of aptamers for clinical applications has focused on their inhibitory effects on the function of the target^5^. Recently, however, various aptamer modulators that alter the target’s response to a stimulus or directly activate a target have been reported. The most common way that aptamers modulate receptor function is through receptor dimerization^6–9^. Some receptors can be activated through artificial dimerization induced by a dimeric aptamer, although the aptamer monomer has no effect on receptor activation. Aptamers can also function as agonists, activating target receptors and having downstream cellular effects that are independent of the intrinsic ligands^10,11^. The binding of an aptamer agonist to its target receptor appears to induce conformational changes similar to those induced by the interaction between the intrinsic ligand and receptor. Moreover, some aptamers specifically recognize the conformational changes in their target receptors that are induced by the binding of intrinsic ligands^12–14^. These aptamers enhance the binding of intrinsic ligands to their target receptors and potentiate downstream signaling by stabilizing the active conformation of ligand-bound receptors. These previous findings suggest that aptamers can regulate target protein functions by inducing or stabilizing conformational changes.

In the present study, we identified a new aptamer modulator, named IR-A62, that binds to the extracellular domain of the insulin receptor (IR). IR-A62 is a biased agonist that preferentially induces Y1150 monophosphorylation of IR and selectively activates glucose uptake without inducing an increase in cellular proliferation. IR-A62 also exhibits mutual binding cooperativity with insulin with respect to its binding to IR, which varies according to the concentrations of the ligands. At low concentrations, IR-A62 and insulin act as positive allosteric modulators (PAMs), promoting the binding of the other to IR. In contrast, at high concentrations, IR-A62 and insulin act as negative allosteric modulators (NAMs), interfering with the binding of the other to IR. Given that the IR forms a stable dimer, in which two monomers are linked by disulfide bonds, these results imply that IR-A62 may bind to the same site on IR as insulin^15^. To our knowledge, the ability of IR-A62 to act as both a PAM agonist and a NAM agonist is very rare among aptamers, antibodies, peptides, and small molecules. Therefore, the present findings suggest that the potential uses of aptamers as target modulators can be extended beyond their roles as simple binders, inhibitors, or agonists.

## Materials and methods

### Reagents and antibodies

Aptamers were synthesized by Aptamer Science, Inc. (Pohang, Korea). 3-Isobutyl-1-methylxanthine (IBMX), dexamethasone, insulin from bovine pancreas, and insulin labeled with FITC were purchased from Sigma–Aldrich (St. Louis, MI, USA). Recombinant versions of the extracellular domain of the insulin-like growth factor (IGF)-1 receptor and human IR were purchased from R&D Systems. T4 polynucleotide kinase was purchased from New England Biolabs (Ipswich, MA, USA). Adenosine triphosphate that was ^32^P-labeled on the gamma-phosphate group was purchased from PerkinElmer. Anti-IR β-subunit (CT-3) antibody was purchased from Santa Cruz Biotechnology (Santa Cruz, CA, USA). Anti-phospho-IR (Y1146), anti-phospho-IR (Y1150), and anti-phospho-tyrosine (4G10) antibodies were purchased from Millipore. Anti-phospho-IR (Y1322), anti-phospho-IR (Y1316), anti-phospho-IR (Y1150/Y1151), and anti-phospho-IR (Y960) antibodies were purchased from Invitrogen (Carlsbad, CA, USA). Anti-phospho-ERK1/2 (T202/Y204), anti-phospho-AKT (T308), and anti-phospho-AKT (S473) antibodies were purchased from Cell Signaling Technology (Danvers, MA, USA). Goat anti-rabbit IgG and anti-mouse IgG secondary antibodies conjugated to DyLight 800 were purchased from Invitrogen.

### In vitro selection of IR aptamers

We performed SELEX to identify IR-specific aptamers, as previously described^14^. Briefly, the single-stranded DNA (ssDNA) library used for SELEX consisted of a 40-mer random region flanked by 20-mer constant regions. The 40-mer random region contained 5-[N-(1-naphthylmethyl)carboxamide]−2’-deoxyuridine (Nap-dU) in place of deoxythymidine (dT) to enhance the hydrophobic interaction between the aptamer and its target. Fifty picomoles of HIS-tagged recombinant extracellular domain of IR (His 28-Lys 944, R&D Systems) were incubated with 100 pmol of the ssDNA library at 37 °C for 30 min in selection buffer (40 mM HEPES (pH 7.5), 102 mM NaCl, 5 mM KCl, 5 mM MgCl_2_, and 0.05% Tween-20). To immobilize the IR proteins, the protein and ssDNA library mixture was incubated with 20 µl of TALON Dynabeads (Invitrogen) at 37 °C for 15 min. To remove unbound ssDNAs, the beads were then washed five times with 100 µl selection buffer. One hundred seventy microliters of 2 mM NaOH solution were added to extract the ssDNA from the IR proteins, and then 160 µl of the eluate was mixed with 40 µl of 8 mM HCl for neutralization. The extracted ssDNAs were then amplified using a 5′-OH-terminal biotinylated reverse primer (IQ5 Multicolor Real-time PCR Detection System, Bio-Rad). To immobilize the biotinylated antisense strands, the amplified DNAs were mixed with 25 µl of MyOne Streptavidin Dynabeads (Invitrogen), and 180 µl of 20 mM NaOH was added to elute the sense strands at 37 °C for 5 min. After discarding the eluted sense strands, the immobilized antisense strands were washed three times with selection buffer. The beads were then incubated with 60 µl extension reaction mix (1× KOD DNA polymerase buffer containing 500 pmol forward primer; 0.0625 U KOD DNA polymerase; 0.5 mM each of dATP, dGTP, dCTP, and Nap-modified dUTP) at 68 °C for 60 min to replace dT with the sense strand containing Nap-dU. After washing three times with 180 µl of selection buffer, 180 µl of 20 mM NaOH was added to elute the sense strands. To neutralize the eluate, 175 µl of the eluate was incubated with 5 µl of 180 mM HEPES and 5 µl of 700 mM HCl. The eluted sense strands were used for the next round of selection, and after eight rounds of SELEX, the enriched ssDNA pool was sequenced.

### Aptamer binding assay

The affinities of aptamers for the extracellular domains of IR (His 28-Lys 944) and IGF-1 receptor (Gln 31­Asn 932) were measured using a filter binding assay. [γ-^32^P]-ATP was used to label the 5′-end of the aptamer (the reaction mix contained 1 μl of 10× T4 polynucleotide kinase buffer, 0.25 μl of 10 U/μl T4 polynucleotide kinase, 0.25 μl of gamma-^32^P-ATP 3,000 Ci/mmol, and 1 pmol of aptamer and was made up to a volume of 10 μl with H_2_O and then incubated at 37 °C for 30 min). To remove unincorporated ATP, the mixture was loaded onto size-exclusion spin columns (MicroSpin G-50 columns, GE Healthcare). To reconstitute the aptamer structure, the mixture was slow-cooled to 37 °C at 0.1 °C/s in binding buffer (40 mM HEPES (pH 7.5), 120 mM NaCl, 5 mM KCl, 5 mM MgCl2, and 0.002% Tween-20) after heating at 95 °C for 3 min. The aptamer was incubated with target proteins at various concentrations for 30 min at 37 °C, and then the aptamer–protein mixture was incubated with 5.5 μl Zorbax silica beads (Agilent) for 1 min with shaking to pull down the aptamer–protein complexes. The beads bound to the aptamer–protein complex were partitioned using nitrocellulose filter plates (Millipore) and washed in the binding buffer to remove the unbound aptamer. The amount of ^32^P labeling the aptamer was measured by exposure to photographic film and quantified using Amersham Typhoon gel and blot imaging systems. The dissociation constant (Kd) of the aptamers was analyzed by fitting the binding data to a one-site saturation equation using SigmaPlot (Systat Software, San Jose, CA, USA).

### Cell culture and adipocyte differentiation

Rat-1 cells overexpressing human IR (Rat-1/hIR) were kindly provided by Dr. Nicholas J. G. Webster of the University of California, San Diego. 3T3-L1 and MCF-7 cells were purchased from the American Type Culture Collection. We used high-glucose Dulbecco’s-modified Eagle’s medium (DMEM) containing 10% (vol/vol) fetal bovine serum (FBS, Gibco) to maintain the MCF-7 cells and Rat-1/hIR cells. High-glucose DMEM containing 10% bovine serum (BS, Gibco) was used to maintain the 3T3-L1 preadipocytes prior to differentiation. All the cells were incubated at 37 °C in a humidified atmosphere containing 5% CO_2_. Before initiating differentiation, 3T3-L1 preadipocytes were cultured for 2 days after reaching confluence. DMEM containing 1 μM dexamethasone, 500 nM IBMX, 850 nM insulin, and 10% FBS was used to stimulate adipocyte differentiation, and after 2 days, this medium was changed to DMEM containing 10% FBS and 850 nM insulin for an additional 2 days. Finally, this medium was replaced with DMEM containing 10% FBS alone, and the cells were incubated for 4–5 days until at least 90% of the cells exhibited lipid droplets.

### Sample preparation for western blotting

To evaluate the phosphorylation of proteins using western blotting, cells were seeded in 12-well plates. For serum starvation, the cells were incubated in a medium lacking FBS for 3 h before insulin or aptamer stimulation. Aptamers and insulin were prepared in Krebs-Ringer HEPES buffer (25 mM HEPES (pH 7.4), 120 mM NaCl, 5 mM KCl, 1.2 mM MgSO_4_, 1.3 mM CaCl_2_ and 1.3 mM KH_2_PO_4_). To reconstitute their tertiary structure, aptamer samples were heated for 5 min at 95 °C and then slowly cooled to room temperature. After stimulation with insulin or aptamer, the cells were washed three times with cold PBS and then lysed in lysis buffer (150 μl/well) (50 mM Tris-HCl (pH 7.4), 150 mM NaCl, 1 mM EDTA, 20 mM NaF, 10 mM β-glycerophosphate, 2 mM Na_3_VO_4_, 1 mM PMSF, 10% glycerol, 1% Triton-X and a protease inhibitor cocktail). The cell lysates were sonicated and centrifuged at 20,000 × *g* for 15 min at 4 °C, and the supernatant was mixed with 5× Laemmli sample buffer. After heating at 95 °C for 10 min, the proteins were separated on Bis-Tris gels and transferred to nitrocellulose membranes. The membranes were incubated in blocking buffer (PBS, 5% nonfat dried milk, and 0.1% NaN_3_) for 30 min at room temperature and then probed with the indicated antibodies at 4 °C overnight. The membranes were then washed three times in TTBS buffer (20 mM Tris, 150 mM NaCl, 0.1% Tween-20) for 10 min each and incubated with secondary antibodies for 1 h at room temperature. After washing the membranes a further three times with TTBS buffer for 10 min each, the intensities of specific bands were analyzed using an LI-COR Odyssey infrared imaging system.

### Flow cytometry

Rat-1/hIR cells were seeded in 100 mm dishes and grown to 70% confluence. To detach the cells without digesting the membrane proteins, we used PBS containing 5 mM EDTA but no trypsin. The detached cells were then incubated in blocking buffer (PBS, 1% BSA, and 0.1% NaN_3_) for 30 min at 4 °C on a rotating shaker at 10 rpm. The cells were then separated into equal aliquots (1 × 10^6^ cells/sample), and FITC-labeled IR-A62 or FITC-labeled insulin was diluted with blocking buffer and mixed with the cells. Unlabeled ligands (insulin or IR-A62 without FITC) were added at the same time as the FITC-labeled ligands. After incubation for 1 h at 4 °C on a rotating shaker at 10 rpm, the cells were washed twice with cold PBS to remove unbound FITC-labeled ligands. They were then fixed with PBS containing 4% paraformaldehyde for 30 min at room temperature, and the binding of FITC-labeled ligands was measured by flow cytometry (BD Biosciences FACS Canto II).

### 2-Deoxy-d-glucose uptake

To measure 2-deoxy-d-glucose uptake, fully differentiated 3T3-L1 adipocytes were prepared in 24-well plates. Before insulin or aptamer stimulation, the 3T3-L1 adipocytes were serum-starved in DMEM without FBS for 3 h. After stimulation with insulin and/or aptamer for the described time, the cells were incubated with 2-deoxy-[^14^C]-glucose (0.1 µCi/ml) for 10 min (500 µl/well) and then washed three times with cold PBS containing 25 mM d-glucose. Subsequently, 500 µl of lysis buffer (0.5 N NaOH and 1% SDS) was added to each well, 450 µl of cell lysate was mixed with 4 ml of a liquid scintillation cocktail (Research Products International), and glucose uptake was measured using a liquid scintillation counter (Hidex 300 SL).

### Cell proliferation assay

MCF-7 breast cancer cells were cultured at 10^4^ cells/well in 24-well plates in DMEM (low glucose (1 g/l), without phenol red and pyruvate) containing 10% FBS. After 24 h, the cells were washed twice with DMEM lacking FBS. Next, the cells were serum-starved in DMEM containing 0.5% FBS for 24 h and stimulated with insulin or IR-A62 aptamer in DMEM containing 0.5% FBS for 72 h, with the medium containing the insulin or IR-A62 aptamer being replaced every 24 h. The cells were then fixed with 4% paraformaldehyde in PBS for 30 min, and the DNA in the cells was stained using 1 µM SYTO 60 in PBS for 1 h. The relative number of cells was then analyzed by measuring the fluorescence of SYTO 60-stained DNA using the LI-COR Odyssey infrared imaging system.

### Effect of the aptamer on the blood glucose level of mice

All of the experimental procedures were performed in accordance with the Animal Care Guidelines of the Laboratory Animal Resource Center of Pusan National University School of Medicine after receiving the approval of the Pusan National University Institutional Animal Care and Use Committee (approval number PNU-2018-1916). C57BL/6 mice were purchased from Koatech (Pyeongtaek, Gyeonggi-do, Korea), and C57BLKS/J-Db/Db and C57BLKS/J-Ob/Ob mice were purchased from Central Lab Animal Inc. (Seoul, Korea). The mice were housed under a 12-h light/dark cycle at 21–23 °C and fed a normal diet (Purina Rodent Chow #38057; Cargill Agri Purina, Seoul, Korea). To establish a model of type I diabetes, C57BL/6 mice were intraperitoneally injected with streptozotocin (STZ, 50 mg/kg in 0.1 M sodium citrate buffer, pH 4.5; Sigma–Aldrich) for 5 consecutive days. Their blood glucose levels were measured weekly using a blood glucose test meter (Accu-Check Active; Roche Diagnostics) after sampling by a tail-vein puncture to confirm the development of hyperglycemia. After 7 weeks of treatment, the mice underwent insulin tolerance testing or aptamer tolerance testing. To determine the effects of insulin and IR-A62 on the blood glucose levels of type I diabetic mice, 1.5 U/kg insulin or 10 mg/kg IR-A62 was injected subcutaneously into the STZ-treated mice. To determine the effects of insulin and IR-A62 on the blood glucose levels of type II diabetic mice, 3 U/kg insulin or 20 mg/kg IR-A62 was injected subcutaneously into C57BLKS/J-Db/Db and C57BLKS/J-Ob/Ob mice. In each instance, the blood glucose levels were measured at the indicated time points.

## Results

### Identification of the IR-A62 aptamer

SELEX was performed to identify aptamers that bind to the extracellular domain of IR (His 28-Lys 944). The single-strand DNA library used contained a 40-mer random region flanked on both sides by 20-mer constant regions that were used for PCR amplification of the library. To improve the specificity and affinity of the aptamer–protein interaction, Nap-dU was used instead of thymine bases in the 40-mer random regions^16^. In this way, we obtained 41 different aptamers containing Nap-dU modifications.

**Fig. 1:**
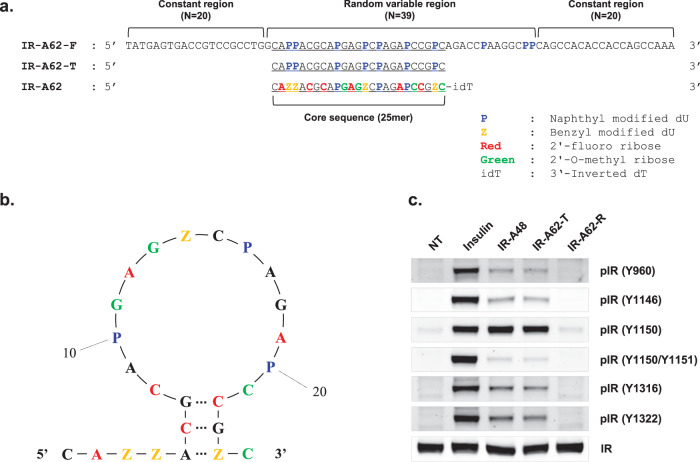
Sequence and agonistic effect on insulin receptor phosphorylation of IR-A62. **a** Comparison of the sequences of IR-A62-F (full length), IR-A62-T (truncated core sequence), and IR-A62 (chemically modified). **b** The secondary structure of IR-A62 was predicted using Mfold software. **c** The effect of IR-A62 on IR phosphorylation. The phosphorylation of six tyrosine residues was analyzed using site-specific anti-phosphotyrosine antibodies. Rat-1/hIR cells were stimulated with 50 nM insulin for 5 min or 200 nM IR-A48, IR-A62, or IR-A62-R for 1 h. ‘IR-A62-R’ is the reverse sequence of IR-A62 (5′-CZGCCPAGAPCZGAGPACGACZZAC-3′).

To evaluate the autophosphorylation of IR induced by the aptamers, Rat-1 cells overexpressing human IR (Rat-1/hIR) were stimulated with 500 nM aptamers for 1 h. We used the IR-A48 agonistic aptamer as a positive control to compare the efficacy of the novel aptamers^11^. Although most of the aptamers had no effect or a smaller effect than the IR-A48 agonistic aptamer on IR autophosphorylation, one aptamer, IR-A62-F, significantly induced IR autophosphorylation to a similar extent to IR-A48. Full-length IR-A62-F is a 79-mer that contains a 39-mer variable region and two 20-mer constant regions (Fig. 1a). Furthermore, we identified a core sequence (IR-A62-T) of IR-A62-F that is essential for its agonistic activity by comparing the effects of IR-A62-F truncation variants containing 3′ or 5′ sequential deletions (data not shown). IR-A62-T consists of 25 nucleotides, of which seven are Nap-dUs, and forms a small stem-loop structure (Fig. 1b). IR-A62-T showed biased agonism, preferentially phosphorylating a specific tyrosine residue of IR, similar to IR-A48 (Fig. 1c). In contrast to insulin, which increased phosphorylation at the Y960, Y1146, Y1150, Y1151, Y1316, and Y1322 residues, IR-A62-T preferentially stimulated the phosphorylation of Y1150, which is in the kinase domain of IR. Moreover, the reversed sequence of IR-A62-T (IR-A62-R) did not stimulate the phosphorylation of Y1150, which indicates that the agonistic effect of IR-A62-T is not caused by a nonspecific interaction with oligonucleotides.

### Post-SELEX optimization of the IR-A62 aptamer

**Fig. 2:**
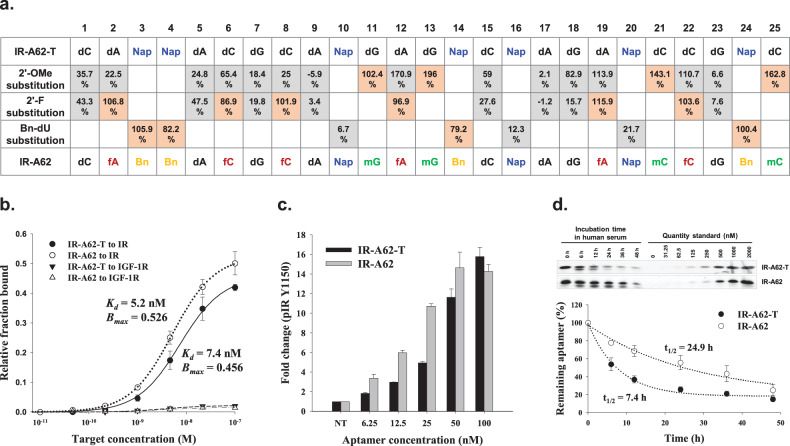
Post-SELEX optimization of IR-A62. **a** Summary of 2’-OMe or 2’-F substitution scans at the A, C, and G positions and Bn-dU substitution scans at the Nap-dU positions (mG: 2’-OMe G, mC: 2’-OMe C, fA: 2’-F A, fC: 2’-F C, Nap: Nap-dU, Bn: Bn-dU). The IR Y1150 phosphorylation induced by the IR-A62-T variants was compared using western blotting. The percentage values represent the Y1150 phosphorylation band intensities associated with the IR-A62-T variants compared to those associated with IR-A62-T. **b** The affinities of IR-A62-T or IR-A62 for the insulin receptor or insulin-like growth factor type 1 receptor were measured using a filter binding assay. The dissociation constant (Kd) was determined by fitting the data to a one-site saturation model. Data are presented as the mean ± standard deviation of two independent replicates. **c** Rat-1/hIR cells were stimulated with various concentrations of IR-A62-T or IR-A62 for 1 h, and then the level of IR Y1150 phosphorylation induced by aptamers was estimated using western blotting. The relative band intensities are presented as the mean ± standard deviation of two independent replicates. **d** In vitro stability of IR-A62-T and IR-A62 in 90% human serum. Aptamer degradation was analyzed using denaturing polyacrylamide gel electrophoresis. Data are presented as the mean ± standard deviation of three independent replicates, and the half-life values were determined by fitting to a one-phase exponential decay model.

A critical limitation of the in vivo use of aptamers is their rapid degradation by serum nucleases^17^. Therefore, it is essential to improve their stability by chemically modifying the nucleotides, such as by adding a methoxy (2’-OMe) or fluoro (2’-F) group at the 2’-sugar position of the ribose. However, such chemical modifications can seriously affect the binding of the aptamer to its target. To determine whether the efficacy of IR-A62-T was affected by the incorporation of modified nucleotides, we prepared IR-A62-T variants in which each dA, dC, and dG nucleotide was substituted by the corresponding 2’-OMe derivative (mA, mC, and mG) (Supplementary Fig. 1a). The effects of the IR-A62-T variants on IR phosphorylation were then evaluated by comparison with IR-A62-T in Rat-1/hIR cells, and the results showed that 11-mG, 12-mA, 13-mG, 19-mA, 21-mC, 22-mC, and 25-mC had no effect or positive effects on the activity of IR-A62-T (Fig. 2a, Supplementary Fig. 1b). We finally chose the 11-mG, 13-mG, 21-mC, and 25-mC modifications to lengthen the distances between each modification because consecutive 2’-OMe modifications significantly disturb the activity of IR-A62-T (Supplementary Fig. 1c). We also performed a similar screen of IR-A62-T variants containing the corresponding 2’-F-derivatives (fA, fC, and fG) in place of each nucleotide, except at the four 2’-OMe modifications sites (Supplementary Fig. 2a). The results showed that 2-fA, 6-fC, 8-fC, 12-fA, 19-fA, and 22-fC had no effect on the activity of IR-A62-T (Supplementary Fig. 2b). To evaluate the combined effects of both the 2’-OMe and 2’-F modifications on IR-A62-T activity, we then tested three IR-A62-T variants containing both 2’-OMe and 2’-F modifications and found that the IR-A62-T variants showed slightly higher activity than the original IR-A62-T (Supplementary Fig. 2c).

The placement of hydrophobic side chains at the 5-position of uracil improves the success of SELEX and increases the affinity of aptamers by adding hydrophobicity to aptamer-target interactions^16^. However, these hydrophobic sites also increase the plasma clearance of the molecules in vivo, which has a negative effect on the pharmacokinetic properties of therapeutic aptamers^18^. Therefore, to reduce the hydrophobicity of IR-A62-T, seven IR-A62-T variants were synthesized, in which each Nap-dU was replaced by 5-(N-benzylcarboxamide)-2’-deoxyuridine (Bn-dU) (Supplementary Fig. 3a). Substitution with Bn-dU at 10-Nap, 16-Nap, and 20-Nap significantly reduced the activity of IR-A62-T (Supplementary Fig. 3b). Therefore, we ultimately selected 3-Bn, 4-Bn, 14-Bn, and 24-Bn as the most appropriate Bn-dU substitutions for IR-A62-T.

The results of the testing of IR-A62-T variants with chemical modifications are summarized in Fig. 2a. The most favorable combination of substitutions was in a derivative named IR-A62, which contained four 2’-OMe groups, six 2’-F groups, four Bn-dU side chains, and three Nap-dU side chains (Fig. 1a, Fig. 2a). The affinity (*K*_*d*_) and maximal binding capacity (*B*_max_) of IR-A62 were slightly superior to those of unmodified IR-A62-T (Fig. 2b). Consistent with the results of the aptamer binding assay, IR-A62 was a more potent inducer of Y1150 phosphorylation on IR than IR-A62-T (Fig. 2c, Supplementary Fig. 4). Moreover, we assessed the nuclease resistance of IR-A62 using an in vitro serum stability assay, in which IR-A62-T and IR-A62 were labeled with a 3′-inverted dT (3′-idT) to protect the aptamers from degradation by 3′-exonucleases in the serum and were incubated with 90% human serum at 37 °C for up to 48 h. The degradation of the aptamers at various time points was then analyzed using denaturing polyacrylamide gel electrophoresis, which demonstrated the stability of IR-A62 (serum half-life [*t*_1/2_]=24.9 h) was significantly superior to that of IR-A62-T (*t*_1/2_ = 7.4 h) (Fig. 2d). These results indicate that the combination of chemical modifications successfully improved the nuclease stability of IR-A62-T without causing any loss of agonistic activity. Therefore, all subsequent experiments were performed using IR-A62 containing these modifications.

### IR-A62 demonstrates binding cooperativity that differs in a concentration-dependent manner

**Fig. 3:**
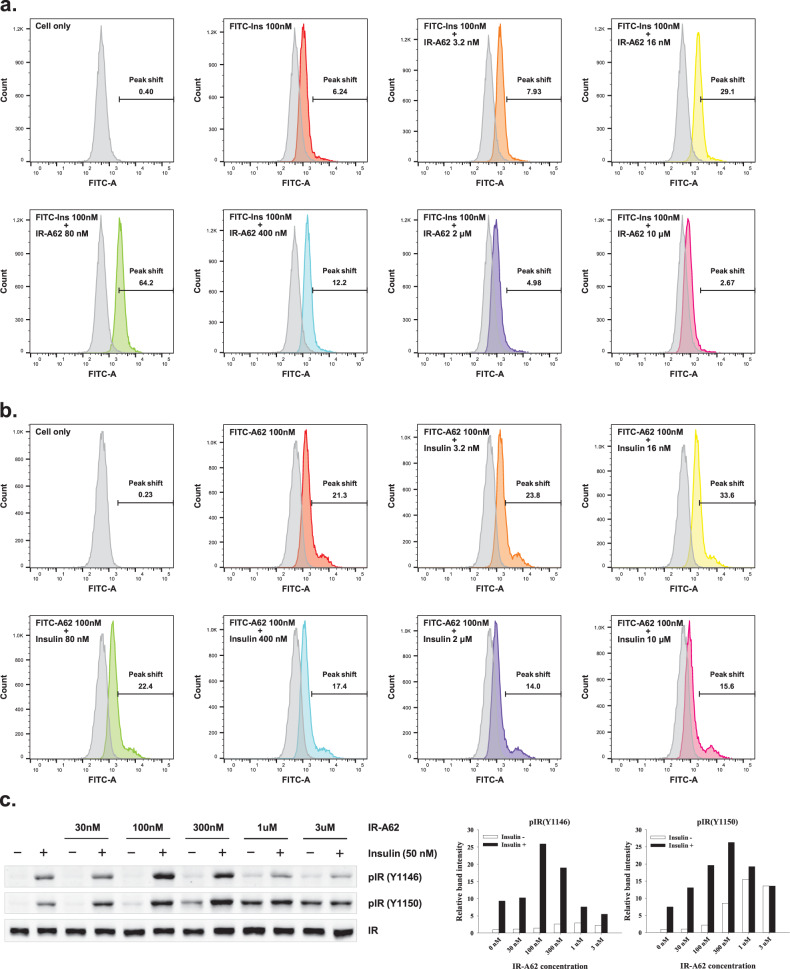
Mutual binding cooperativity of insulin and IR-A62. Rat-1/hIR cells were incubated with **a** 100 nM FITC-labeled insulin (FITC-Ins) and various concentrations of IR-A62 or **b** 100 nM FITC-labeled IR-A62 (FITC-A62) and various concentrations of insulin. To analyze the binding of insulin or IR-A62, the fluorescence generated by FITC was measured using flow cytometry. **c** IR phosphorylation resulting from costimulation using insulin and IR-A62. Rat-1/hIR cells were incubated with 50 nM insulin and various concentrations of IR-A62 for 5 min, and then IR phosphorylation was estimated using western blotting.

To determine whether the binding site of IR-A62 is allosteric or orthosteric, we next studied the effect of IR-A62 on the binding of insulin to IR on the plasma membrane. Rat-1/hIR cells were incubated with FITC-labeled insulin (100 nM) and various concentrations of IR-A62 (3.2 nM, 16 nM, 80 nM, 400 nM, 2 µM, or 10 µM), and insulin binding was measured using flow cytometry. FITC-labeled insulin alone caused a 6.24% shift in the peak fluorescent intensity (Fig. 3a). At low IR-A62 concentrations (3.2–80 nM), coincubation of FITC-labeled insulin with IR-A62 gradually increased the peak shift, up to 64.2%. However, as the concentrations of IR-A62 were increased further (400 nM–10 µM), the binding of FITC-labeled insulin gradually decreased to 2.67%, which was a lower level than with FITC-labeled insulin alone.

To verify that the binding cooperativity between insulin and IR-A62 varies depending on concentration, we also measured the binding of FITC-labeled IR-A62 to IR in the presence of various concentrations of insulin (3.2 nM, 16 nM, 80 nM, 400 nM, 2 µM, and 10 µM). Consistent with the results of the insulin-binding assay, coincubation with low insulin concentrations (3.2–16 nM) gradually increased FITC-labeled IR-A62 binding compared to incubation with FITC-labeled IR-A62 alone (Fig. 3b). Moreover, as the concentration of insulin was further increased (80 nM–10 µM), the binding of FITC-labeled IR-A62 gradually decreased. These results imply that insulin and IR-A62 cooperatively bind in a concentration-dependent manner. At low concentrations, insulin and IR-A62 act as mutual PAMs, with the binding of one promoting the binding of the other to IR. However, at high concentrations, IR-A62 and insulin act as mutual NAMs, inhabiting each other’s binding to IR.

In a previous study, we demonstrated that the enhancement of insulin binding to IR by a PAM aptamer potentiates the phosphorylation of tyrosine residues in the intracellular domain of IR^14^. As shown in Fig. 1c, insulin binding to IR leads to the autophosphorylation of tyrosine residues, and IR-A62 preferentially induces monophosphorylation of the Y1150 residue of IR. Thus, we can distinguish between insulin- or IR-A62-induced IR phosphorylation by comparing the levels of phosphorylation of Y1150 and other tyrosine residues. To determine whether the concentration-dependent cooperativity between insulin and IR-A62 affects IR autophosphorylation, we evaluated the phosphorylation of IR in the presence of 50 nM insulin and various concentrations of IR-A62 (30 nM, 100 nM, 300 nM, 1 µM and 3 µM). The IR Y1146 phosphorylation induced by insulin increased at low IR-A62 concentrations (30–300 nM) and decreased at higher IR-A62 concentrations (1–3 µM). Because IR Y1146 phosphorylation is induced by insulin but not by IR-A62, this implies that insulin-induced IR phosphorylation can be potentiated or inhibited by concentration-dependent cooperativity with IR-A62 (Fig. 3c). Although a low level of IR Y1150 phosphorylation was induced by IR-A62 alone at low IR-A62 concentrations (30–100 nM), the level induced by coincubation with insulin and IR-A62 was significantly higher. However, as the IR-A62 concentration was increased further (300 nM–3 µM), the IR Y1150 phosphorylation induced by coincubation with insulin and IR-A62 gradually decreased to a similar level to that induced by 3 µM IR-A62 alone. These findings demonstrate that the concentration-dependent differences in the mutual cooperativity displayed by insulin and IR-A62 directly affect the autophosphorylation of IR.

### IR signaling is induced by IR-A62

**Fig. 4:**
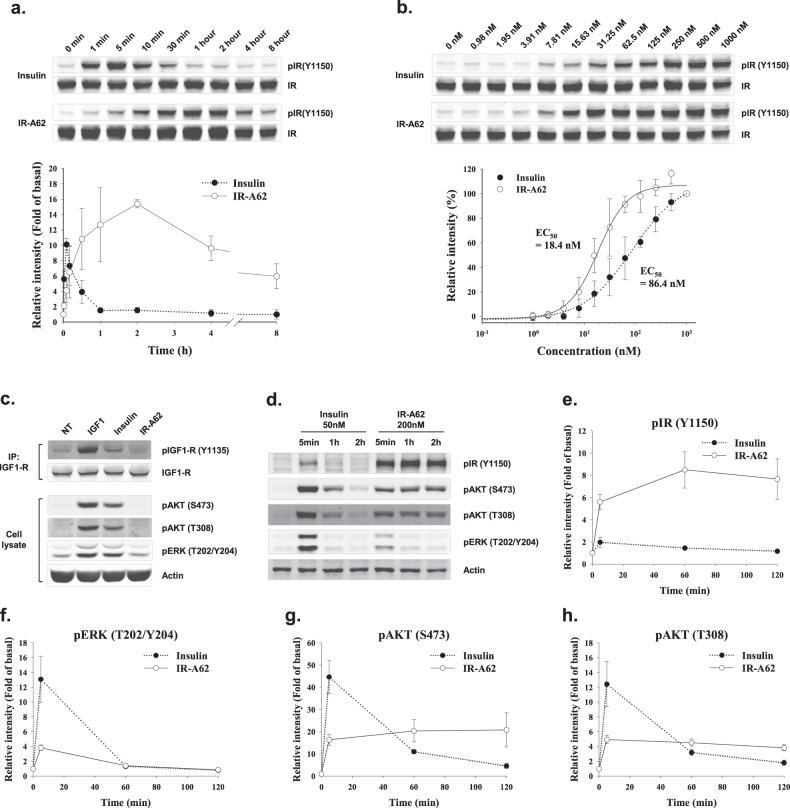
Effects of the dose and duration of treatment with IR-A62 on IR Y1150 phosphorylation and downstream signaling. **a** IR Y1150 phosphorylation was measured following the incubation of Rat-1/hIR cells with 100 nM insulin or 100 nM IR-A62 for 1 min, 5 min, 10 min, 30 min, 1 h, 2 h, 4 h, or 8 h. The relative band intensities are presented as the mean ± standard deviation of two independent replicates. **b** Rat-1/hIR cells were incubated with various concentrations of insulin for 5 min or IR-A62 for 1 h. The relative band intensities are presented as the mean ± standard deviation of two independent replicates. To determine the EC50, the data were fitted to a four-parameter logistic equation. **g** HeLa cells were incubated with 50 nM insulin-like growth factor-1 for 10 min, 100 nM insulin for 10 min, or 1 µM IR-A62 for 1 h. IGF-1R was then immunoprecipitated to assess its phosphorylation. **d** Fully differentiated 3T3-L1 adipocytes were incubated with 50 nM insulin or 200 nM IR-A62 for 5 min, 1 h, or 2 h. The phosphorylation kinetics of **e** IR Y1150, **f** extracellular signal-related kinase (ERK) T202/Y204, **g** AKT S473, and **h** AKT T308 are presented as the mean ± standard deviation of three independent replicates.

We have shown that IR-A62 is a biased agonist that preferentially induces Y1150 phosphorylation of IR, similar to IR-A48 (Fig. 1c). Moreover, in our previous study, we showed that IR-A48 is characterized by slower and more sustained phosphorylation kinetics of IR and downstream proteins than insulin^11^. To further investigate the signaling kinetics of IR-A62, we first compared the kinetics of the Y1150 phosphorylation of IR induced by insulin and IR-A62. In contrast to insulin, IR-A62 slowly increased the phosphorylation of IR at Y1150 over 2 h, and this phosphorylation was sustained for 8 h (Fig. 4a), which indicates that IR-A62 also induces signaling slowly but sustains it over a relatively long period of time. However, IR-A62 had a 4.7-fold lower EC_50_ (18.4 nM) for IR phosphorylation (Y1150) than insulin (86.4 nM) (Fig. 4b). Furthermore, IR-A62 did not bind to IGF-1 receptor (IGF-1R), despite the high degree of structural similarity between IR and IGF-1R (Fig. 2b). Consistent with this binding specificity, IR-A62 had no effect on the phosphorylation of IGF-1R (Fig. 4c).

To characterize the downstream signaling activated by IR-A62, we treated fully differentiated 3T3-L1 adipocytes with IR-A62 for 5 min, 1 h, or 2 h and measured the phosphorylation of IR, AKT, and extracellular signal-regulated kinase (ERK) (Fig. 4d). IR-A62 (200 nM) stimulation for 5 min only slightly increased the phosphorylation of ERK, and the level of AKT phosphorylation induced by IR-A62 was lower than that induced by insulin, even though the level of IR Y1150 phosphorylation induced by IR-A62 was significantly higher than that induced by insulin (Fig. 4e–h). Moreover, the AKT phosphorylation induced by IR-A62 was sustained for up to 2 h, but the ERK phosphorylation induced by IR-A62 was not. Taken together, these results imply that although IR-A62 induces IR Y1150 phosphorylation more potently than insulin, its effects on signaling downstream of IR are minor and less than those of insulin. However, the activation of the AKT pathway by IR-A62 was sustained over a longer period of time than the activation induced by insulin, which is consistent with the Y1150 phosphorylation kinetics of IR.

### The effects of IR-A62 on glucose uptake and cell proliferation

**Fig. 5:**
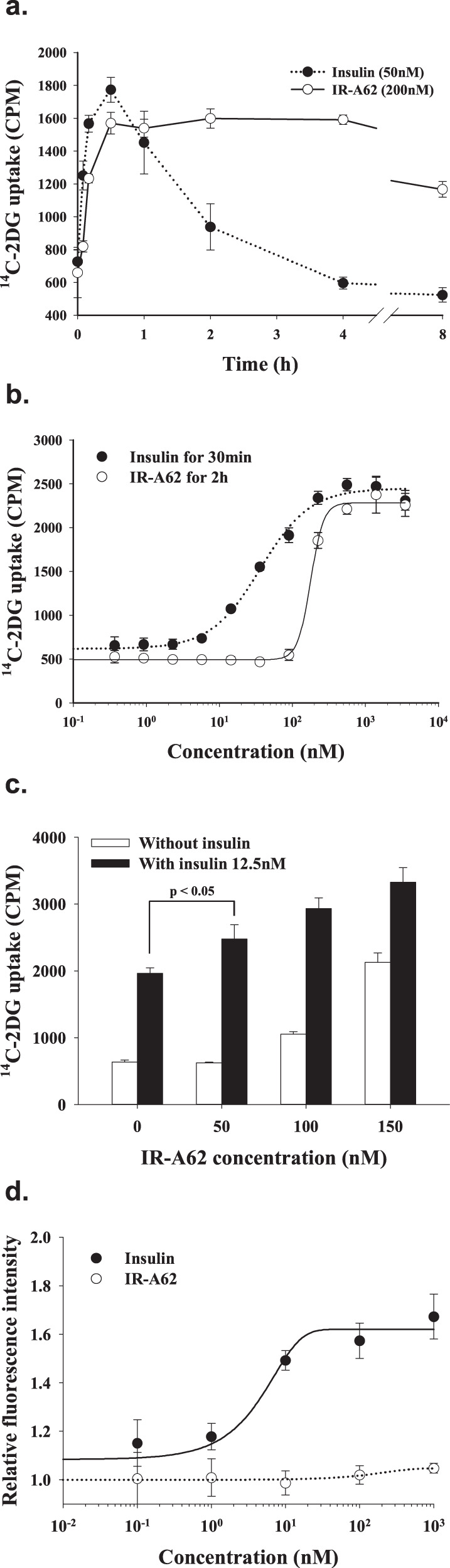
IR-A62 selectively stimulates glucose uptake but not cell proliferation. **a** To measure 2-deoxy-d-glucose uptake, fully differentiated 3T3-L1 adipocytes were incubated with 50 nM insulin or 200 nM IR-A62 for the indicated periods of time. **b** Fully differentiated 3T3-L1 adipocytes were incubated with various concentrations of insulin and IR-A62 for 30 min or 2 h, respectively. **c** Fully differentiated 3T3-L1 adipocytes were treated with 50 nM, 100 nM, or 150 nM IR-A62 in the absence or presence of 12.5 nM insulin for 30 min. Data are presented as the mean ± standard deviation of three biological replicates. *P* values were determined using one-way ANOVA followed by Tukey’s multiple comparisons test. **d** MCF-7 breast cancer cells were incubated with various concentrations of IR-A62 or insulin for 72 h. Cell proliferation was quantified by measuring the amount of SYTO 60-stained DNA using an LI-COR Odyssey infrared imaging system. Data are presented as the mean ± standard deviation of two independent replicates.

IR is a critical regulator of metabolic processes, such as glucose uptake, fat synthesis, gluconeogenesis, and glycogenolysis^19^. Many previous studies have shown that the metabolic effects of insulin and IR mainly involve the AKT pathway rather than the MAPK pathway^20^. Because IR-A62 stimulated AKT phosphorylation in 3T3-L1 adipocytes, we next quantified the time- and dose-dependent effects of IR-A62 on 2-deoxy-glucose uptake. The timing of the effect of IR-A62 on glucose uptake was similar to the timing of its effect on IR and AKT phosphorylation (Fig. 5a): glucose uptake increased slowly over 30 min, in contrast to the rapid effect of insulin, and was sustained for up to 4 h. Moreover, in contrast to the glucose uptake following insulin stimulation, which decreased rapidly after 30 min, the glucose uptake induced by IR-A62 remained greater than half-maximal after 8 h.

To compare the potency of the effects of IR-A62 and insulin on glucose uptake, we next measured 2-deoxy-glucose uptake in 3T3-L1 adipocytes after stimulation with various doses of each (Fig. 5b). The maximal glucose uptake induced by insulin or IR-A62 at concentrations >500 nM did not differ significantly. However, similar to the effects of each on the phosphorylation of AKT at S473 and T308, the glucose uptake induced by insulin was higher than that induced by IR-A62 at low concentrations (5–100 nM). Moreover, IR-A62 exponentially increased glucose uptake at concentrations of 100–500 nM (Hill coefficient: 4.9) compared with insulin, which increased glucose uptake more gradually in the range 5–500 nM (Hill coefficient: 1.27). Consequently, although IR-A62 has a lower EC_50_ value for IR Y1150 phosphorylation than insulin, the EC_50_ value of IR-A62 for glucose uptake was higher (177.6 nM) than that of insulin (36.5 nM). These results indicate that IR-A62 alone increases glucose uptake to a level that is comparable with the effect of insulin.

In Fig. 3, we show that the cooperative binding of insulin and IR-A62 to IR is mutual and depends on the concentration of each. To determine whether the effect of IR-A62 on increasing insulin binding by IR-A62 potentiates glucose uptake, we measured the glucose uptake induced by IR-A62 in the absence or presence of insulin. Figure 5b shows that glucose uptake in the presence of IR-A62 began to increase at ~100 nM. Therefore, 50 nM, 100 nM, and 150 nM IR-A62 were used to stimulate 3T3-L1 adipocytes in the absence or presence of a low concentration of insulin (12.5 nM) to determine the effect of IR-A62 on insulin-induced glucose uptake (Fig. 5c). Fifty nanomolar IR-A62 alone did not induce glucose uptake, but cotreatment with insulin potentiated insulin-induced glucose uptake. In addition, the glucose uptake induced by IR-A62 alone was greater at concentrations of 100 nM or 150 nM than 50 nM, and the level of glucose uptake that was induced by insulin and IR-A62 together was greater than that induced by a combination of 50 nM IR-A62 and insulin. These results indicate that IR-A62 cooperatively increases glucose uptake when coadministered with insulin.

Insulin is also a growth factor: it induces the proliferation and growth of cancer cells, principally via the MAPK pathway^21^. In contrast to insulin, IR-A62 had little effect on the MAPK pathway (Fig. 4d, f). However, we also performed a cell proliferation assay in MCF-7 human breast cancer cells to determine whether IR-A62 affected cell proliferation (Fig. 5d). In this assay, insulin-stimulated cell proliferation by up to 1.76-fold, but IR-A62 did not significantly change the number of cells, even at a concentration of 1 µM. Given that the glucose uptake induced by IR-A62 was maximal at ~500 nM, this implies that IR-A62 is a biased agonist that selectively induces the metabolic effects of IR, similar to IR-A48.

### IR-A62 reduces glycemia in diabetic mice

Our in vitro data demonstrate that IR-A62 is a biased agonist that induces glucose uptake but not cellular proliferation. Moreover, IR-A62 is stable when exposed to serum nucleases in vitro (*t*_1/2_ = 24.9 h). Therefore, to investigate the effect of IR-A62 on blood glucose in vivo, we compared the effects of subcutaneous injections of insulin or IR-A62 on the blood glucose levels of diabetic mice.

**Fig. 6:**
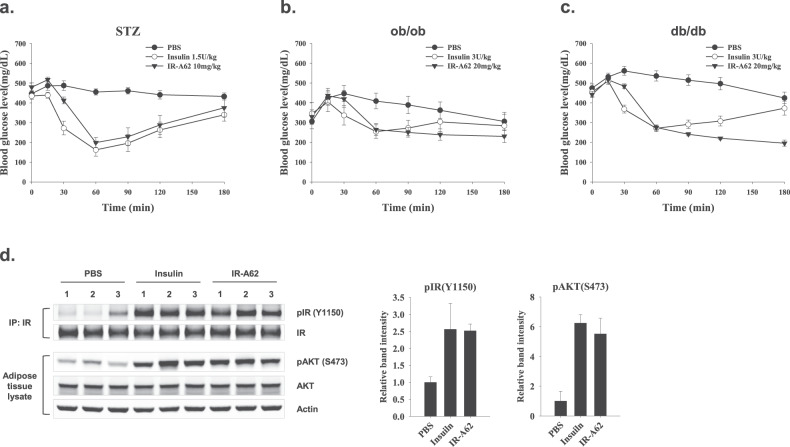
IR-A62 administration reduces the blood glucose levels of diabetic mice. **a** Streptozotocin-treated mice were subcutaneously administered vehicle (PBS), insulin 1.5 U/kg or 10 mg/kg IR-A62. **b** *ob*/*ob,* and **c** *db*/*db* mice were subcutaneously administered vehicle (PBS), insulin 3 U/kg, or IR-A62 20 mg/kg. Data are presented as the mean ± standard deviation (*n* = 6 mice/group). **d** Effect of IR-A62 on IR and AKT phosphorylation in adipose tissue. Normal mice were subcutaneously administered vehicle (PBS), insulin 1.5 U/kg or 10 mg/kg IR-A62. The adipose tissues were collected 30 min after administration. Data are presented as the mean ± standard deviation (*n* = 3 mice/group).

We established a model of type 1 diabetes by administering STZ to C57BL/6 mice, in which the basal glucose levels were maintained at ~450 mg/l (Fig. 6a). The subcutaneous injection of either insulin or IR-A62 markedly reduced the blood glucose levels within 1 h, and these gradually returned to their basal levels over the next 2 h. The kinetics and magnitudes of the effects of insulin and IR-A62 on the blood glucose levels did not differ significantly.

Next, we administered insulin or IR-A62 subcutaneously to *ob*/*ob* and *db*/*db* mice, which are well-established models of type 2 diabetes^22^. Both insulin and IR-A62 markedly reduced their blood glucose levels within 1 h (Fig. 6b, c), but the blood glucose levels of mice administered insulin returned to their resting levels within the following 3 h, whereas those of mice administered IR-A62 did not. These results imply that IR-A62 lowers blood glucose to a similar extent to insulin, but the kinetics of its effects on blood glucose differ according to the mouse model used.

To determine whether IR-A62 increases IR and AKT phosphorylation in peripheral tissues in the same way as insulin, we administered insulin (1.5 U/kg) or IR-A62 (10 mg/kg) subcutaneously to normal C57BL/6 mice (*n* = 3). After 30 min, adipose tissue samples were collected, and AKT (S473) and IR (Y1150) phosphorylation were measured (Fig. 6d). Consistent with the in vitro findings, both insulin and IR-A62 increased AKT and IR phosphorylation. This implies that the ability of IR-A62 to reduce glucose in vivo may be the result of the activation of IR in peripheral tissues.

## Discussion

In this study, we identified a new agonistic aptamer, IR-A62, which induces the phosphorylation of IR by binding to the extracellular domain of IR. IR-A62 also preferentially stimulates Y1150 monophosphorylation in the kinase domain of IR. This is a unique property of agonistic aptamers for IR because insulin induces the phosphorylation of all six tyrosine residues. Moreover, the effects of IR-A62 on insulin binding depend on the ligand concentration. At low concentrations, IR-A62 acts as a PAM, potentiating both insulin binding and IR phosphorylation. Conversely, at high concentrations, IR-A62 acts as a NAM, inhibiting both insulin binding and IR phosphorylation. Because IR-A62 alone acts as an agonist and activates IR, the classification of IR-A62 as a PAM agonist and a NAM agonist depends on the concentration at which it is used. The cooperativity of IR-A62 is also mutual with insulin: insulin likewise enhances or inhibits the binding of IR-A62 to IR in a concentration-dependent manner. To our knowledge, this variable cooperativity between IR-A62 and insulin, which depends on the concentration of each, represents a phenomenon that has not been reported to date.

The biased agonism of IR-A62 is similar to that described for IR-A48, another agonistic aptamer for IR^11^. These two aptamer agonists preferentially stimulate the Y1150 monophosphorylation of IR. Moreover, their selectivity for downstream signaling to metabolic endpoints is also identical. Both IR-A62 and IR-A48 stimulate AKT phosphorylation and glucose uptake but have little effect on ERK phosphorylation or cellular proliferation. However, one critical difference between IR-A62 and IR-A48 lies in their binding properties. IR-A48 is an allosteric modulator that exerts its effects independent of insulin binding, whereas IR-A62 and insulin exhibit binding cooperativity that depends on their concentrations.

The effects of IR-A62 on insulin binding are somewhat similar to those of the IR-A43 aptamer^14^. IR-A43 alone cannot stimulate IR phosphorylation but acts as a mutual PAM with insulin. It enhances not only insulin binding to IR but also IR phosphorylation, downstream signaling, and insulin-stimulated glucose uptake. The binding site of IR-A43, which was identified by IR mutation studies, is an allosteric site that is distinct from the insulin binding site. However, IR-A62 is not identical to IR-A43, in that it competitively binds insulin at high concentrations. Thus, IR-A62 has complex effects that seem to be a mixture of those of IR-A48, IR-A43, and a NAM agonist.

We speculate that the unique cooperativity demonstrated by IR-A62 is dictated by the structure of IR. The activity of IR-A62 as a PAM means that IR-A62 binds to an allosteric site distant from the insulin-binding site of IR. Moreover, the NAM activity of IR-A62 implies that the binding of IR-A62 to IR may be competitive with insulin. These two apparently contradictory conclusions can be explained by the fact that IR is a dimer that has two insulin-binding sites. In contrast to other members of the receptor tyrosine kinase family, which exist as monomers when not binding ligands, IR always exists as a dimer that is stably linked by disulfide bonds^23^. Inactive IR, in the absence of insulin, has a symmetrical inverted V-shaped structure^24,25^. Although one IR dimer has two insulin-binding sites, the binding of one molecule of insulin to a receptor dimer can initiate dimer activation^26^. One insulin molecule forms a complex with the leucine-rich repeat 1 (L1) and α-helical C-terminal domains of IR, which alters the receptor conformation to an asymmetric inverted L-shape structure. Moreover, at high insulin concentrations, up to four insulin molecules can bind to a receptor dimer^27^. The binding of two or more insulin molecules to the receptor dimer causes the formation of a T-shaped structure because of the translocation of both L1 domains toward the FnIII-1 domains. Therefore, one plausible model for the effects of IR-A62 is that one insulin and one IR-A62 molecule bind to each insulin binding site of a receptor dimer at low ligand concentrations. The binding of insulin and IR-A62 may confer structural stability on the ligand–receptor complex, which may reduce the dissociation of the bound ligands. However, as the concentration of insulin or IR-A62 increases, the ratio of IRs in which two insulin or two IR-A62 molecules bind to both insulin-binding sites of a receptor dimer to the total number of IRs increases. Therefore, at a high concentration of insulin or IR-A62, each competes with the other ligand bound to the opposite binding site, thereby interfering with each other’s binding. To confirm the veracity of this model, further structural studies of the IR-A62-IR complex are needed. We also expect that such structural studies will help further elucidate the mechanism of IR activation.

Consistent with the results of the in vitro glucose uptake assay, we have shown that subcutaneous IR-A62 administration reduces the glycemia of diabetic mice to the same degree as insulin over 1 h. Furthermore, the serum insulin concentration may be important for the in vivo activity of IR-A62. In STZ-treated mice, the reduction in blood glucose induced by IR-A62 was gradually reversed after 1 h, whereas it lasted up to 3 h in *ob*/*ob* and *db*/*db* mice. The effects of insulin on blood glucose slowly dissipated after 1 h in all three mouse models of diabetes, which may be explained by the mutual binding cooperativity of IR-A62 and insulin. One of the important differences among the STZ-treated, *ob*/*ob,* and *db*/*db* mice is their serum insulin concentrations. STZ reduces the serum insulin concentration by causing the necrosis of pancreatic beta cells, but in *ob*/*ob* and *db*/*db* mice, overweight and insulin resistance result in significant hyperinsulinemia^28^. Given that IR-A62 and insulin show mutual binding cooperativity for IR, a plausible explanation is that the binding of IR-A62 to the IR in *ob*/*ob* and *db*/*db* mice is rendered more stable by the positive cooperativity of the high serum insulin concentration than in STZ-treated mice. The long-lasting blood-glucose-lowering effect of IR-A62 that was identified in vivo suggests that IR-A62 has potential as an addition to or a substitute for long-acting insulin in the treatment of diabetes.

For many years, only the binding of most aptamers to their targets has been studied. However, recent studies have shown that aptamers can regulate the activities of their targets. Aptamers can potentiate the binding and activity of intrinsic ligands by recognizing specific conformations of target receptors^12–14^. Moreover, an allosteric aptamer can activate its target receptor and initiate biased signaling without the need for the intrinsic ligand^11^. The most important differences between aptamers and antibodies are that aptamers are much smaller and recognize the surface structure of the target protein^4,29^. Therefore, we predict that aptamers can bind to various sites on the target protein, thereby inducing complex conformational changes or, conversely, stabilizing target proteins in specific conformations. It remains difficult to explain the complex properties of IR-A62 because structural analysis of receptor modulation by aptamers has not been reported to date. However, we speculate that IR-A62 may induce changes in the structure of the IR that differ from those induced by insulin. We believe that the present findings will suggest new directions for aptamer research and use. The discovery and further characterization of aptamers with unique properties, such as IR-A62, may expand their potential for use as target modulators that have differing effects on antibodies.

## Supplementary information

Supplementary Fig. 1,2,3,4
