# Supplementary material for: An aptamer agonist of the insulin receptor acts as a positive or negative allosteric modulator, depending on its concentration
Source: Exp Mol Med. 2022 Apr 27;54(4):531–41. doi: 10.1038/s12276-022-00760-w (PMC9076861; doi:10.1038/s12276-022-00760-w)
Supplement: Supplementary file 1 — Supplementary Fig. 1,2,3,4 [file 12276_2022_760_MOESM1_ESM.pdf]

Supplementary Fig. 1

a.

2'-OMe variants	sequence
#1 1-mC	mCA66ACGCA6GAG6C6AGA6CCG6C
#2 2-mA	CmA66ACGCA6GAG6C6AGA6CCG6C
#3 5-mA	CA66mACGCA6GAG6C6AGA6CCG6C
#4 6-mC	CA66AmCGCA6GAG6C6AGA6CCG6C
#5 7-mG	CA66ACmGCA6GAG6C6AGA6CCG6C
#6 8-mC	CA66ACGmCA6GAG6C6AGA6CCG6C
#7 9-mA	CA66ACGCmA6GAG6C6AGA6CCG6C
#8 11-mG	CA66ACGCA6mGAG6C6AGA6CCG6C
#9 12-mA	CA66ACGCA6GmAG6C6AGA6CCG6C
#10 13-mG	CA66ACGCA6GAmG6C6AGA6CCG6C
#11 15-mC	CA66ACGCA6GAG6mC6AGA6CCG6C
#12 17-mA	CA66ACGCA6GAG6C6mAGA6CCG6C
#13 18-mG	CA66ACGCA6GAG6C6AmGA6CCG6C
#14 19-mA	CA66ACGCA6GAG6C6AGmA6CCG6C
#15 21-mC	CA66ACGCA6GAG6C6AGA6mCCG6C
#16 22-mC	CA66ACGCA6GAG6C6AGA6CmCG6C
#17 23-mG	CA66ACGCA6GAG6C6AGA6CCmG6C
#18 25-mC	CA66ACGCA6GAG6C6AGA6CCG6mC

b.

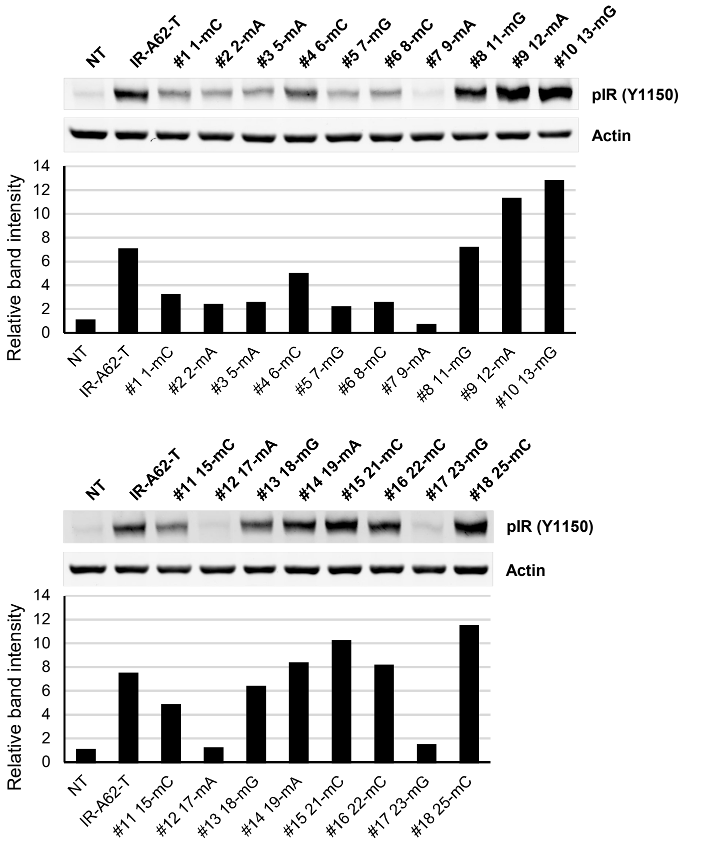

c.

2'-OMe combination	Sequence
Com #1	CA66ACGCA6mGmAmG6C6AGA6mCCG6C
Com #2	CA66ACGCA6mGAmG6C6AGA6mCCG6C
Com #3	CA66ACGCA6GmAmG6C6AGA6mCCG6C
Com #4	CA66ACGCA6GAmG6C6AGmA6mCCG6C
Com #5	CA66ACGCA6GAmG6C6AGA6mCmCG6C
Com #6	CA66ACGCA6GAmG6C6AGA6mCCG6mC
Com #7	CA66ACGCA6GAmG6C6AGA6mCCG6C
Com #8	CA66ACGCA6mGAmG6C6AGmA6mCCG6mC

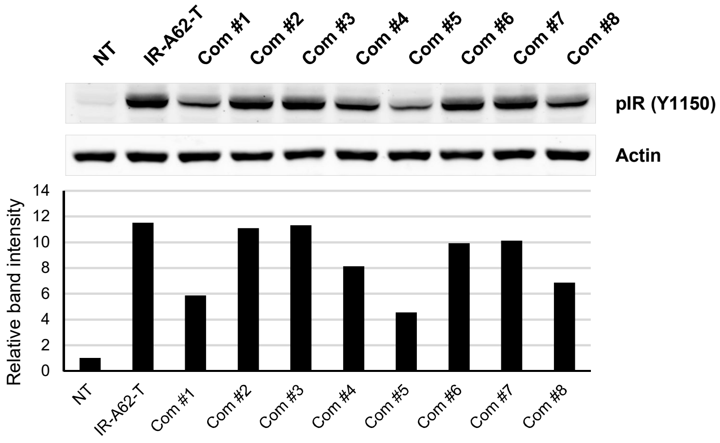

Supplementary Fig. 1 (a) Sequences of IR-A62-T variants in which each dA, dC and dG nucleotide was substituted by the corresponding 2'-OMe-derivative (mA, mC and mG). (b) The level of IR Y1150 phosphorylation by the IR-A62-T variants containing 2'-OMe substitution was esmimated in Rat-1/hIR cells using western blotting. (c) Combinations of 11-mG, 12-mA, 13-mG, 19-mA, 21-mC, 22-mC and 25-mC. The level of IR Y1150 phosphorylation by the combinations was esmimated in Rat-1/hIR cells.

Supplementary Fig. 2

a.

2'-F variants	sequence
#1 1-fC	fCA66ACGCA6GAG6C6AGA6CCG6C
#2 2-fA	CfA66ACGCA6GAG6C6AGA6CCG6C
#3 5-fA	CA66fACGCA6GAG6C6AGA6CCG6C
#4 6-fC	CA66AfCGCA6GAG6C6AGA6CCG6C
#5 7-fG	CA66ACfGCA6GAG6C6AGA6CCG6C
#6 8-fC	CA66ACGfCA6GAG6C6AGA6CCG6C
#7 9-fA	CA66ACGCfA6GAG6C6AGA6CCG6C
#8 12-fA	CA66ACGCA6GfAG6C6AGA6CCG6C
#9 15-fC	CA66ACGCA6GAG6fC6AGA6CCG6C
#10 17-fA	CA66ACGCA6GAG6C6fAGA6CCG6C
#11 18-fG	CA66ACGCA6GAG6C6AfGA6CCG6C
#12 19-fA	CA66ACGCA6GAG6C6AGfA6CCG6C
#13 22-fC	CA66ACGCA6GAG6C6AGA6CfCG6C
#14 23-fG	CA66ACGCA6GAG6C6AGA6CCfG6C

b.

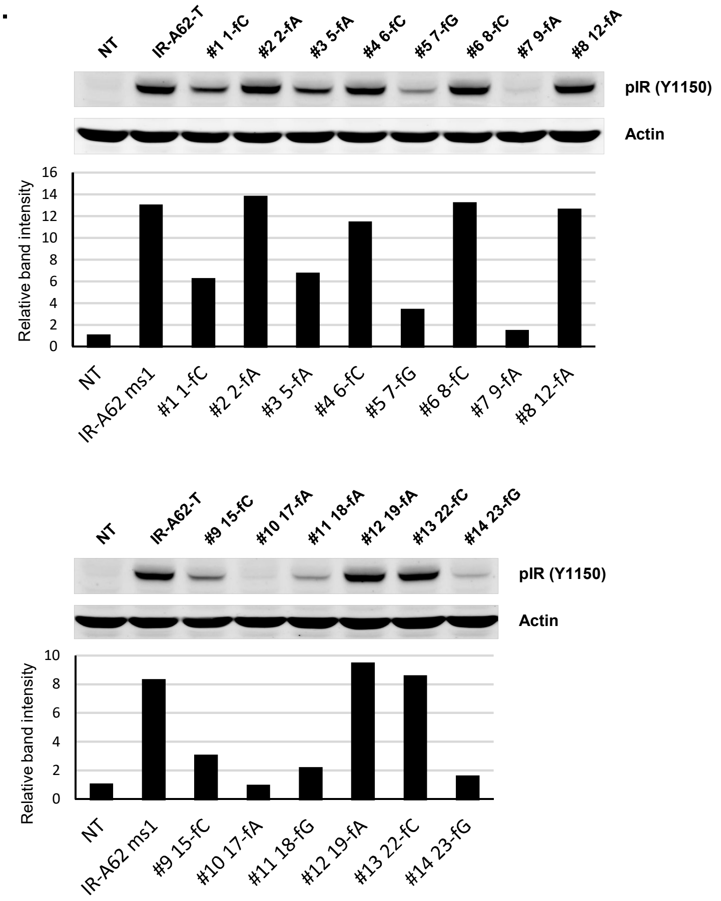

c.

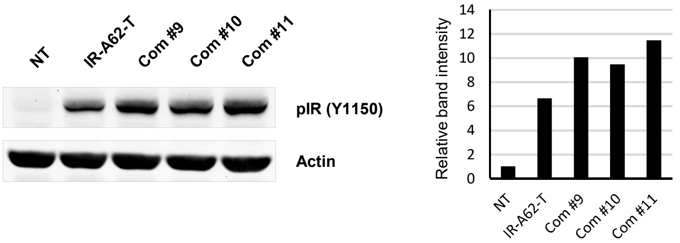

2'-OMe + 2'-F combination	Sequence
Com #9	CfA66AfCGfCA6mGfAmG6C6AGA6mCCG6mC
Com #10	CA66ACGCA6mGAmG6C6AGfA6mCfCG6mC
Com #11	CfA66AfCGfCA6mGfAmG6C6AGfA6mCfCG6mC

Supplementary Fig. 2 (a) Sequences of IR-A62-T variants containing the corresponding 2'-F-derivatives (fA, fC and fG) in place of each nucleotide. (b) The level of IR Y1150 phosphorylation by the IR-A62-T variants containing 2'-F substitution was esmimated in Rat-1/hIR cells using western blotting. (c) Combinations of 11-mG, 12-mA, 13-mG, 19-mA, 21-mC, 22-mC, 25-mC, 2-fA, 6-fC, 8-fC, 12-fA, 19-fA and 22-fC. The level of IR Y1150 phosphorylation by the combinations was esmimated in Rat-1/hIR cells using western blotting.

Supplementary Fig. 3

a.

Bz variants	sequence
#1 3-Bz	CAZPACGCAPGAGPCPAGAPCCGPC
#2 4-Bz	CAPZACGCAPGAGPCPAGAPCCGPC
#3 10-Bz	CAPPACGCAZGAGPCPAGAPCCGPC
#4 14-Bz	CAPPACGCAPGAGZCPAGAPCCGPC
#5 16-Bz	CAPPACGCAPGAGPCZAGAPCCGPC
#6 20-Bz	CAPPACGCAPGAGPCPAGAZCCGPC
#7 24-Bz	CAPPACGCAPGAGPCPAGAPCCGZC

b.

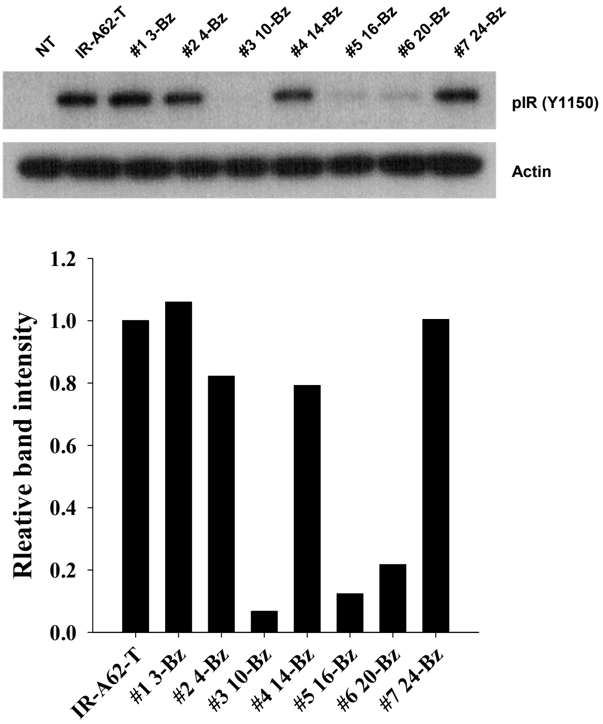

Supplementary Fig. 3 (a) Sequences of IR-A62-T variants in which each Nap-dU was replaced by Bn-dU. (b) The level of IR Y1150 phosphorylation by the IR-A62-T variants containing Bn-dU substitution was esmimated in Rat-1/hIR cells using western blotting.

Supplementary Fig. 4

Replicate #1

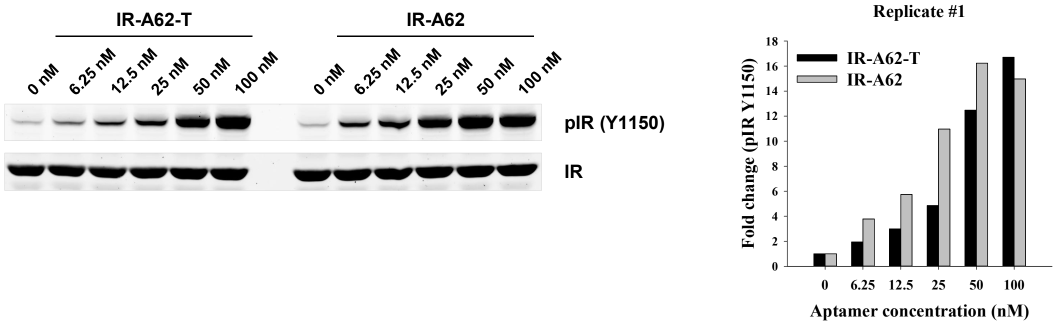

Replicate #2

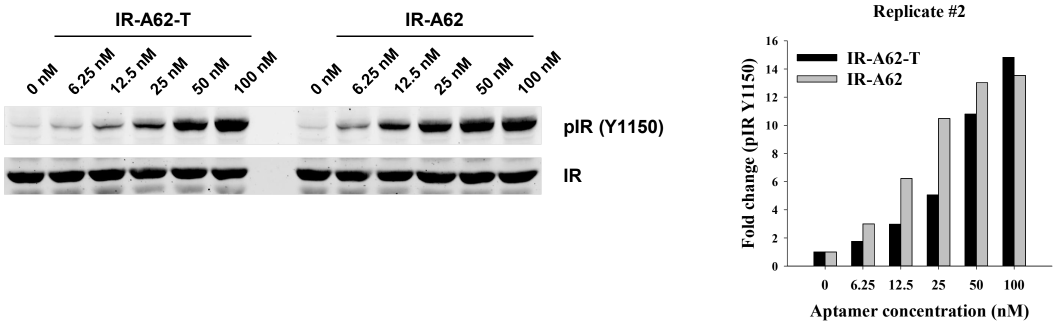

Supplementary Fig. 4 Comparison of concentration-response between unmodified IR-A62-T and fully modified IR-A62. Rat-1/hIR cells were stimulated with various concentrations of IR-A62-T or IR-A62 for 1 h, and the level of IR Y1150 phosphorylation was esmimated using western blotting. These experiments were independently replicated.
